# Mental health outcomes in times of economic recession: a systematic literature review

**DOI:** 10.1186/s12889-016-2720-y

**Published:** 2016-02-03

**Authors:** Diana Frasquilho, Margarida Gaspar Matos, Ferdinand Salonna, Diogo Guerreiro, Cláudia C. Storti, Tânia Gaspar, José M. Caldas-de-Almeida

**Affiliations:** 1 Medical School, CMDT, Nova University Lisbon, Lisbon, Portugal; 2 Faculty of Human Kinetics, ISAMB, University of Lisbon, Lisbon, Portugal; 3 Institute of Active Lifestyle, Faculty of Physical Culture, Palacky University Olomouc, Olomouc, Czech Republic; 4 Psychiatry Department, Faculty of Medicine, University of Lisbon, Lisbon, Portugal; 5 European Monitoring Centre for Drugs and Drug Addiction, Lisbon, Portugal; 6 Aventura Social/ISAMB, University of Lisbon and Lisbon Lusíada University, Lisbon, Portugal; 7 Department of Mental Health, Medical School, Nova University Lisbon, Lisbon, Portugal

**Keywords:** Economic recession, Mental disorders, Mental health, Substance-related disorders, Suicide, Unemployment

## Abstract

**Background:**

Countries in recession experience high unemployment rates and a decline in living conditions, which, it has been suggested, negatively influences their populations’ health. The present review examines the recent evidence of the possible association between economic recessions and mental health outcomes.

**Methods:**

Literature review of records identified through Medline, PsycINFO, SciELO, and EBSCO Host. Only original research papers, published between 2004 and 2014, peer-reviewed, non-qualitative research, and reporting on associations between economic factors and proxies of mental health were considered.

**Results:**

One-hundred-one papers met the inclusion criteria. The evidence was consistent that economic recessions and mediators such as unemployment, income decline, and unmanageable debts are significantly associated with poor mental wellbeing, increased rates of common mental disorders, substance-related disorders, and suicidal behaviours.

**Conclusion:**

On the basis of a thorough analysis of the selected investigations, we conclude that periods of economic recession are possibly associated with a higher prevalence of mental health problems, including common mental disorders, substance disorders, and ultimately suicidal behaviour. Most of the research is based on cross-sectional studies, which seriously limits causality inferences. Conclusions are summarised, taking into account international policy recommendations concerning the cost-effective measures that can possibly reduce the occurrence of negative mental health outcomes in populations during periods of economic recession.

## Background

Economic recessions have been estimated to significantly affect the population’s health and wellbeing, which applies, in particular, to vulnerable groups of people [1–5]. In countries that have been hardest hit by the latest recession, which started in 2007, the living and working conditions have substantially worsened [6]. Work became more precarious and unemployment rates increased as a result of the slowdown in global growth and consequent deterioration of the labour markets [7]. For instance, almost half of the citizens of Europe reported knowing someone who had lost his/her job as a direct result of the crisis [8]. Rates of involuntary part-time employment have also been rising since the beginning of the recession [9]. Overall, people are more fearful about losing their employment [8] since competition for jobs is rising and finding work quickly is perceived as unlikely. It is estimated that labour markets will take time to improve even though there are prospects for economic recovery [6]. Levels of poverty and social exclusion have worsened, mainly in groups that were already at risk [10]. During this recession, more people have been reporting being at risk of being unable to cope with unexpected expenses and even facing difficulties with paying ordinary bills or buying food over the coming year [8].

It is known that the health of populations is shaped by the socioeconomic context, welfare systems, labour markets, public policies, and demographic characteristics of countries [4]. There are strong reasons to believe that changes in these key determinants may be reflected in the mental wellbeing of populations [11]. Therefore, mental health should be a health area regarded as possibly vulnerable during a recession [12], especially if mental disorders were already highly prevalent even before the crisis began [13]. Nonethless, some authors have argued that associations between contracting economies and levels of well-being may show mixed patterns of both positive and negative impacts [14]. However, this current recession is likely to aggravate and boost mental health problems through growing socioeconomic risk factors such as unemployment, financial strain, debts, and job-related problems [3]. People facing these major life changes are more prone to mental ill-health [15–18]. It has also been theorised that economic pressure and unemployment have a devastating impact on families, in particular children, since the family is the most important context for their healthy development [19, 20].

This paper intends to cover the main sources and types of recent evidence on populations’ mental health outcomes in times of economic recession. Specifically to summarize the mental health outcomes and the socioeconomic determinants most frequently addressed by the literature on economic recessions, which groups of people seem to be the most vulnerable, and to determine possible research needs.

## Methods

### Search strategy and definition of terms

A systematic search was performed in Medline, PsycINFO, SciELO, and EBSCO Host. The keywords used for reference tracing were derived from Medical Subject Headings (MeSH) in combination with key terms used in other reviews [2, 3, 5, 21, 22].

Two sets of keywords were then used and combined: 1) Recession and socioeconomic terms – “Economic recession” OR “Financial crisis” OR “Recession” OR “Unemployment” OR “Socioeconomic deprivation”; combined with 2) Mental health outcomes – “Mental health” OR “Mental disorders” OR “Suicide” OR “Substance-Related Disorders”.

Regarding the recession and socioeconomic terms, besides the logical use of the words “economic recession” and its synonyms, the word “unemployment” was used as it is a widely recognised countercyclical variable, i.e. a phenomenon that increases in recessions [23]. The term “socioeconomic deprivation” was used, on one hand because it is a broad term that includes the characteristics of both social and economic vulnerability that are expected to increase in periods of recession [24], and on the other hand, because of its indisputable negative effect on health [5, 11]. Concerning the mental health outcomes, in addition to “mental health”, the term “mental disorders” was used because it is a MeSH term that encompasses “all psychiatric illness or diseases manifested by breakdowns in the adaptational process expressed primarily as abnormalities of thought, feeling, and behaviour producing either distress or impairment of function”. Although “mental disorders” is a broad term, it does not include suicide, which is known to be associated with major mental health problems [25]. Therefore a specific keyword for that was entered. The term “substance-related disorders” was also included, because using the broad term “mental disorders” did not retrieve papers with clear specific results and this was a MeSH term used in other reference works [26].

### Eligibility criteria and data extraction

Two reviewers independently screened all the titles and abstracts. The final articles in this review are a consensual reflection of both reviewers. They only considered studies for inclusion that were original research papers, peer-reviewed, published between 2004 and 2014, written in English or Portuguese, and showing associated results between recession or socioeconomic terms and mental health outcomes. Moreover, the authors excluded all duplicates, small sample investigations (<1000 except for case–control studies) for precision reasons and strength of effect sizes [27], research that did not employ validated instruments or used an inappropriate methodology regarding the associations under consideration (e.g. ambiguous variables under study, poor construct validity, and drawing of conclusions without statistical support), and qualitative research. The data extraction from each study was based on the following variables: the setting and country, the sample (N and age), the years examined, the mental health outcome(s) and the socioeconomic determinant(s), and the key associations or effects found. In general, we found significant disparities in the methods, data collection procedures, analyses, and contexts of existing studies that complicated direct comparison of results among studies. Because of this diversity of metrics and outcome variables, it was impossible to apply statistical criteria to the studies and for that reason it was not appropriate to perform meta-analysis of the results.

### Mental health outcomes associated with economic recessions

We organised the main results by mental health outcomes and the socioeconomic determinants most frequently addressed by the literature, based on the quality of study design (cohort, case–control, cross-sectional and ecological). The mental health outcomes were clustered into four main groups: 1) psychological wellbeing (measured by continuous variables of mental health distress, self-rated health, and wellbeing or quality of life variables); 2) common mental disorders (assessed by caseness for depression, anxiety, and somatoform disorders); 3) problems related to substance-related disorders (reports on smoking, patterns of alcohol consumption, drug use, and substance-related harms), and 4) reports on suicidal behaviours (suicide mortality, parasuicidal behaviour, suicidal ideation, and attempts). The socioeconomic determinants retrieved were clustered into three groups by: 1) inter-time variables (pre- and post-economic recession changes); 2) macroeconomic indicators (rates of unemployment, GDP, home foreclosure rates), and 3) individual-level indicators (employment status, psychosocial job quality and security, household income, perceived financial strain or security, perceived economy/recession stress, deprivation, indebtedness, housing payment problems, socioeconomic status).

## Results

### Study selection results

**Fig. 1:**
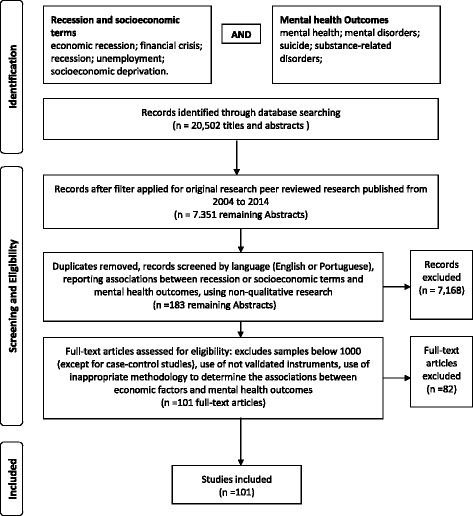
Flow diagram of the multistep selection method

At the beginning, 20,502 studies were identified and were first filtered on the basis of being original peer-reviewed research papers and published between 2004 and 2014. The remaining 7351 papers were then screened by two independent reviewers through their titles and abstracts, and the subsequent filtering was performed on the basis of the following inclusion criteria: not being duplicates, written in English or Portuguese, were non-qualitative research, and reported associations between recession or socioeconomic terms and mental health outcomes. The full texts of 183 studies were then analysed. The number of papers excluded was a consequence of combinations of search keywords such as “crisis” and “mental health” or “suicide” that resulted in papers not relevant to the study objective. From the analysis of the 183 full texts, the investigators further excluded studies that used an inappropriate methodology regarding the associations between economic recession and mental health outcomes, including non-validated instruments, or used small samples (<1000, with the exception of case–control studies). After the previously described multistep selection method, 101 papers were used for the present review. Figure 1 shows the progress of selection for the study and the number of articles at each selection stage.

### Research designs

Table 1 summarizes the main features of the retrieved studies. Two studies used case–control design, 30 were cohort studies, 40 were cross-sectional studies or repeated cross-sectional studies, one used a mixed cross-sectional and case–control design, and 28 were ecological studies.

**Table 1:** Summary of the main features of retrieved studies

Studies research design	
Case–control	2
Cohort	30
Cross-sectional	41
Ecological	28
Samples	
Cross-national population samples	16
National population samples	66
Regional/community samples	19
Geographical allocation of studies’ samples	
EU	61
North America (USA and Canada)	18
Australasia (Australia and New Zealand)	7
Asia (China, Japan, South Korea)	6
South America (Argentina and Brazil)	2
Multicentre	7

### Samples and geographical allocation

More than half (66) of the total of 101 studies used national population samples. Out of these 66, 58 were general population samples and the rest were focusing on specific populations. Two used working populations, two used unemployed populations, two used samples of adolescents, one used a sample of patients attending primary care centres, and one used a sample of older adults. Furthermore, 16 studies used cross-national population samples, of which 11 were general population samples and 5 focused only specific populations. Two studies were samples of children and adolescents; one was a sample of working population; one used a sample of patients attending primary care centres, and one used samples of older adults. An additional 19 studies used community or regional samples. Out of these 19, 10 studies used general population, 3 used samples of workers, 1 used a sample of unemployed adults, 2 used samples of adolescents, 1 used a sample of children and parents, 1 used a sample of older adults, and 1 used a sample of hospital patients.

In terms of geographical allocation, 61 studies were conducted in Europe (7 studies were cross-European, 2 studies used samples from both Greece and Poland, and both Denmark and Sweden, 15 used samples from the UK, 8 from Greece, 7 from Sweden, 7 from Spain, 4 from Italy, 3 from Finland, 2 from Iceland, and 1 population sample each from France, Germany, Hungary, Portugal, Slovakia, and Slovenia). Eighteen studies have North American population samples (15 from USA and 3 from Canada); 7 studies were from Australasia countries (five Australian and two from New Zealand). Two studies were South American, one from Argentina and Brazil, and six studies were from Asian countries (three from South Korea, two from Japan, and one study from Hong Kong). In addition, there were seven multicentre studies that used cross continent population samples from various countries.

### Pre and post-economic recession changes in psychological wellbeing

Studies comparing the data to pre-recession periods show a consistent aggravation of the mental health status of the populations involved (Table 2).

**Table 2:** Characteristics of studies included in the review comparing the data to pre-recession periods and mental health outcomes, 2004–2014

Study	Setting	Study design	N Year Age	Socioeconomic determinants	Mental health outcomes	Associations/Effects	Strengths	Limitations
[28]	National population sample, Greece	Cohort	17,713 (2008–2013) Mean age 39.41y (SD = 8.83)	Inter-time Variables Psychosocial/economic indicators Pre- and post- recession period Employment Status	Psychological Well-being Mental health (CES-D scale) Self-rated health	In the period 2008–2013 unemployed people faced more impaired health (3.21 vs 2.48, t = 8.34, *p* = 0.00) and mental health than did employed people (12.67 vs 9.39, t = 12.28, *p* =0.00). These health differences between unemployed and employed individuals were smaller in 2008–2009 than in 2010–2013.	Temporal order of exposures, confounders, and the outcome under consideration affected all participants at the same time, producing stronger causal conclusions. The results indicate a relationship between unemployment and health/mental health.	The impact of economic recessions varies across cultures and time periods; thus, the generalisability of the findings may be considerably limited by the uniqueness of the Greek situation.
[29]	National population sample, Italy	Cohort	37,782 (2006–2010) 15–64 y	Inter-time Variables Psychosocial/economic indicators Pre- and post- recession period Employment Status	Psychological Well-being Self-rated health	Temporary workers, first job seekers and unemployed individuals all perceive their health as being worse than permanent workers do. The health inequalities between permanent workers and the unemployed rose, especially for males and young people, after the economic recession.	The temporal order of the exposures, confounders, and the outcome under consideration affected all the participants at the same time, producing stronger causal conclusions.	The impact of economic recessions varies across cultures and time periods; thus, generalising findings may be reasonably limited by the uniqueness of the Italian social system.
[31]	National population samples from Greece and Poland	Repeated cross-sectional Case–control	54,120 cases 136,952 controls (2006–2009)	Inter-time Variables Pre- and post-recession period	Psychological Well-being Self-reported health	Relative to the control population (Poland), Greece experienced a significantly bigger increase in the odds of poor health after the crisis (OR = 1.16; 95 % CI 1.04–1.29)	This study benefits from having a control group and cross-national design. The study is composed of comparable surveys across two time points, before and after the onset of the recession.	Its cross-sectional design removes the possibility of causal inference. Data is derived from 2006–2009 and the crisis started in 2008 so the long-term effects of the recession could not be investigated.
[30]	National population sample, Greece	Repeated cross-sectional	10,572 (2006) (2011) >18 years	Inter-time Variables Pre- and post- recession period	Psychological Well-being Self-reported health	Self-reported good health deteriorated from 71 % in 2006 to 68.8 % in 2011 (*P* < 0.05).	The study is composed of comparable surveys across two time periods (pre- and post-recession).	Its cross-sectional design removes the possibility of causal inference. It is limited to 2011.
[32]	National population sample, England, UK	Repeated cross-sectional	106,985 (1991) (2010) 25–64 y	Inter-time Variables Pre- and post- recession period	Psychological Well-being Mental health distress (GHQ-12)	Age-sex adj GHQ-12 caseness increased from 13.7 % (95 % CI:12.9–14.5 %) in 2008 to 16.4 % (95 % CI:14.9–17.9 %) in 2009 and 15.5 % (95 % CI:14.4–16.7 %) in 2010. Women only had a greater prevalence from 1991 until the recession, but men showed an increase over the period.	Uses a continuous measure of mental health symptoms. Large nationally representative dataset surveyed two times.	Its cross-sectional design removes the possibility of causal inference. Limited period of time; the long-term effects of the recession could not be investigated.
[33]	National population sample, Spain	Repeated cross-sectional	23,760 (2006) 16,616 (2012)	Inter-time Variables Psychosocial/economic indicators Pre- and post- recession period Employment Status	Psychological Well-being Mental health distress (GHQ-12)	Results found an increase in the prevalence of poor mental health among men (prevalence ratio = 1.15, 95 % CI 1.04–1.26], especially among those aged 35–54 years, and a slight decrease for women between 2006/07 and 2011/12. There was a larger impact among the unemployed.	The study is composed of comparable surveys across two time points before and after the economic recession period. Representative sample	Its cross-sectional design removes the possibility of causal inference. Limited period of time (2012); the long-term effects of the recession could not be investigated.
[34]	Regional population sample, Working-age women, Stockholm, Sweden	Repeated cross-sectional	27,994 (2006) 22,639 (2010) 18–64 y	Inter-time Variables Psychosocial/economic indicators Pre- and post- recession period Employment Status	Psychological Well-being Mental health distress (GHQ-12)	Mental distress increased among women of all types of employment status between 2006 and 2010, but more so among unemployed women, OR 2.65 (CI 95 % 2.17–3.23) in 2006 and OR 2.81 (CI 95 % 2.20–3.58) in 2010.	The study is composed of comparable surveys across multiple time points before and after the economic recession period.	Its cross-sectional design removes the possibility of causal inference. Data is derived from 2006–2010 and the crisis started in 2008, so the long term effects of the recession could not be investigated. The sample is composed only of women.
[35]	National population sample, Japan	Repeated cross-sectional	168,801 (1986–1989) 150,016 (1998–2001) 20–60 y	Inter-time Variables Psychosocial/economic indicators Pre- and post- recession period Employment status/Income	Psychological Well-being Self-reported health	The OR for poor self-rated health (95 % CI) among middle-class people compared with the highest class was 1.02 (0.92–1.14) before the crisis and increased to 1.14 (1.02–1.29) after the crisis (*p* = 0.02). The association was stronger among males. Unemployed people were twice as likely to report poor health.	The study is composed of comparable surveys across multiple time points before and after the economic recession period, showing reports of increased poor health across all socioeconomic statuses.	Its cross-sectional design removes the possibility of causal inference. The study lacks individual-level information on job insecurity, work overload, or pay cuts that can work as confounders. The outcome was self-reported.
[36]	National population sample, Iceland	Cohort	9807 (2007) 5439 (2009)	Inter-time Variables Psychosocial/economic indicators Pre- and post-recession period Employment Status	Common Mental Disorders Psychological stress (PSS-4)	Age-adj stress levels increased between 2007 and 2009 (*P* = 0.004), only for women (*P* = 0.003). The OR for high stress levels increased only among women (OR = 1.37), especially those who were unemployed (OR = 3.38), students (OR = 2.01), with middle levels of education (OR = 1.65), or in the middle income bracket (OR = 1.59).	This study examines the longitudinal interrelations between employment status and socio-demographic in psychological stress levels during a period of extensive macroeconomic changes.	The impact of economic recessions varies across cultures and time periods; thus, the generalisability of the findings may be reasonably limited by the uniqueness of Icelandic culture, as well as the nature of the 2008 economic collapse in Iceland.
[37]	National population sample, Greece	Repeated cross-sectional	2197 (2008) 2256 (2011) 18–69 y	Inter-time Variables Psychosocial/economic indicators Pre- and post- recession period Perceived financial strain	Common Mental Disorders Depression (SCID-I)	The odds of major depression were greater in 2011 than in 2008 (OR = 2.6, 95 % CI = 1.97–3.43). Financial strain independently and significantly predicts the presence of major depression (OR = 1.2, 95 % CI = 1.13–1.24).	The study is composed of comparable surveys across two time points before and after the period of the economic recession.	Its cross-sectional design removes the possibility of causal inference. Limited period of time; the long-term effects of the recession could not be investigated. Telephone survey.
[38]	National population sample, Greece	Repeated cross-sectional	2197 (2008) 2192 (2009) 18–69 y	Inter-time Variables Psychosocial/economic indicators Pre- and post- recession period Financial strain	Common Mental Disorders Major depressive episode–MDE (SCID-I)	The one-month prevalence of MDE in 2009 was found to be 6.8 %, compared to rates of 3.3 % in 2008 (*p* < 0.0001). Respondents facing serious economic hardship were at higher risk of developing an MDE.	Representative samples and comparable surveys across two time points before and during the period of the economic recession in Greece.	No causal inference can be made because of the cross-sectional nature of the study. The generalisability of the findings is limited by the uniqueness of the 2008 economic collapse in Greece. Limited period of time; the long-term effects of the recession could not be investigated.
[39]	National population sample, patients attending primary care centres, Spain	Repeated cross-sectional	7640 (2006–07) 5876 (2010–11)	Inter-time Variables Psychosocial/economic indicators Pre- and post-recession period Employment status, Mortgage payments	Common Mental Disorders Substance Disorders Depression Anxiety Somatoform Alcohol-related disorders	Since the pre-crisis period (2006), major depression increased by 19.4 %, anxiety by 8.4 %, somatoform disorders by 7.3 %, and alcohol-related disorders by 4.6 %. The risk of depression when unemployed was OR = 2.12, *p* < 0.001. The risk of depression resulting from mortgage payment difficulties was OR = 2.95, *p* < 0.001.	The study is composed of comparable surveys across multiple time points before and after the economic recession period.	Its cross-sectional design removes the possibility of causal inference. Limited period of time; the long-term effects of the recession could not be investigated. Only patients attending and able to access primary care were investigated.
[40]	Regional working population sample, Alberta, Canada	Repeated cross-sectional	3579 (2008–2009)	Inter-time Variables Psychosocial/economic indicators Pre- and post-recession period	Common Mental Disorders Major depressive disorder (MDD) Dysthymia Anxiety (CIDI)	The 12-month prevalence of major depressive disorder (MDD) before September 1, 2008; between September 1, 2008, and March 1, 2009; and between March 1, 2009, and October 30, 2009, was 5.1, 6.8, and 7.6 % (*P* = 0.03), respectively. The lifetime prevalence of dysthymia reported during the 3 periods was 0.4, 0.7, and 1.5 % (*P* = 0.006), respectively. No changes in the 12-month prevalence of social phobia, panic disorder, and generalized anxiety disorder were found over time.	This study examines changes in the population prevalence of common mental disorders before and during the period of the economic recession.	No causal inference can be made because of the cross-sectional nature of the study. The effects of socioeconomic variables were not adjusted. The long-term effects of the recession could not be investigated.
[41]	National population sample, Hong Kong, China	Repeated cross-sectional	3016 (2007) 2011 (2009) 15–65 y	Inter-time Variables Psychosocial/economic indicators Pre- and post-economic crisis period Socioeconomic/Employment Status	Common Mental Disorders Major depressive episode (MDE)	The 12-month prevalence of MDE was significantly higher in 2009 (/12.5 %) than 2007 (8.5 %). The prevalence of MDE in the unemployed group increased from 2007 (14.6 %) to 2009 (17.8 %).	The study is composed of comparable surveys across two time points before and after the period of the economic recession.	Because of population characteristics and cultural norms concerning response to economic adversity, the findings may not generalise to other societies. Its cross-sectional design removes the possibility of causal inference. The long-term effects of the recession could not be investigated.
[42]	National population sample, USA	Cohort	2,050,431 (2006–2007; 2008–2009) >18 y	Inter-time Variables Psychosocial/economic indicators Pre- and post-recession period Employment status	Substance-Disorders Alcohol use	The prevalence of any alcohol use significantly declined from 52.0 % in 2006–2007 to 51.6 % in 2008–2009. There was an increase in the prevalence of frequent bingeing, from 4.8 % in 2006–2007 to 5.1 % in 2008–2009 (*P* < 0.01). Unmarried non-Black men under 30 years who recently became unemployed were at the greatest risk of frequent bingeing.	Large representative sample. Longitudinal measures on changes of alcohol use prevalence during a period of economic recession.	The generalisability of the findings may be reasonably limited to the country’s own policy regarding alcohol use and the social welfare system. Limited period of time; the long-term effects of the recession could not be investigated.
[43]	Community sample, province and city of Buenos Aires, Argentina	Cross-sectional	1000 (2002) 18–65 y	Inter-time Variables During recession period	Substance-Disorders Patterns of drinking behaviour Drinking-related problems (Genacis)	During the economic crisis people drank more at home or at friends’ homes. A large number of respondents also reported that people had changed to cheaper or lower-quality alcoholic drinks.	Brings evidence on how the economic crisis is possibly resulting in increased drinking of cheaper or lower-quality alcoholic drinks.	No causal inference can be made because of the cross-sectional design. The validity of self-reports of sensitive behaviours, such as alcohol consumption. Because of the uniqueness of the Argentinean economic collapse and societal characteristics, the generalisability of the findings may be reasonably limited.
[44]	National population sample, Sweden	Cohort	4,224,210 (1992–1996)	Inter-time Variables Psychosocial/economic indicators Pre- and post-recession period Employment status	Suicidal Behaviours Excess mortality effects (suicide)	During the recession there was no additional risk of mortality as a result of suicide. During the post-recession period, there was an additional risk of mortality through suicide for unemployed men (HR = 1.43; 95 % CI = 1.31, 1.56) but not unemployed women.	This study examines longitudinal changes in suicide mortality during a period of mass unemployment in Sweden. Reports post-recessionary increases on suicide among unemployed men, considering possible time-lagged effects.	This research study fails to determine if mental health declined as a result of unemployment, or loss of benefits or income over time. The generalisability of the findings may be limited by the uniqueness of the Swedish welfare system and its economic recession.
[45]	National population sample, Greece	Repeated cross-sectional	2192 (2009) 2256 (2011)	Inter-time Variables Psychosocial/economic indicators Pre- and post-recession period Financial strain (Index of Personal Economic Distress)	Suicidal Behaviours Suicidal ideation Suicide attempts	The rate of suicidal ideation increased from 5.2 % in 2009 to 6.7 % in 2011 (*χ* ^2^ = 3.92, df = 1, *p* = 0.04). The increase was significant in men (7.1 vs. 4.4 %, *χ* ^2^ = 6.41, df = 1, *p* = 0.011) and those aged 55–64 years (7.2 vs. 1.9 %, *χ* ^2^ = 14.41, df = 1, *p* < 0.001), while it decreased in those younger than 24 years (4.9 vs. 13.9 %, *χ* ^2^ = 15.83, df = 1, *p* < 0.001). Suicidal ideation increased among psychotropic medication users (22.7 vs. 4.5 %, *χ* ^2^ = 11.10, df = 1, *p* < 0.001) and those asking for mental healthcare (17.3 vs. 8.3 %, *χ* ^2^ = 13.36, df = 1, *p* < 0.001). No unemployed respondent reported a suicide attempt in 2009, while the proportion was 4.4 % in 2011 (*χ* ^2^ = 4.12, df = 1, *p* = 0.042).	Representative sample. This study provides evidence on the increase in the prevalence of suicidal ideation and reported suicide attempts in a country facing a deep economic recession.	Reported suicidal ideation and suicide attempts can be susceptible to recall bias or to reluctance on the part of respondents to disclose such sensitive information. No causal inference can be made because of the cross-sectional nature of the study. Limited period of time; the long-term effects of the recession could not be investigated. The generalisability of the findings may be reasonably limited by the uniqueness of the 2008 economic collapse in Greece.
[46]	National population sample, South Korea	Repeated cross-sectional	27,745 (1998) 27,413 (2001) 25,487 (2005) 3335 (2007) >19 y	Inter-time Variables Psychosocial/economic indicators Pre- and post-recession period Socioeconomic inequalities	Common Mental Disorders Suicidal Behaviours Depression (doctor-diagnosed) Suicidal ideation Suicide attempts	The pro-rich inequalities in the prevalence of depression, suicidal ideation and suicide attempts doubled between 1998 and 2007. The CI for depression decreased from −0.126 (SE: 0.068) in 1998 to −0.278 (SE: 0.068) in 2001 and stayed constant. The CI for suicidal ideation fell gradually: −0.138 (SE: 0.012) in 1998 and −0.250 (SE: 0.028) in 2007. The CI for suicide attempts increased from −0.221 (SE: 0.062) in 1998 to −0.175 (SE: 0.075) in 2001 and −0.179 (SE: 0.089) in 2005, and in 2007 to −0.400 (SE: 0.116).	Nationally representative survey data sets The study is composed of comparable surveys across several time points before and after the period of the economic recession.	Reported suicidal ideation and suicide attempts can be susceptible to recall bias or to reluctance on the part of respondents to disclose such sensitive information. Depressed individuals in lower income groups might have been under-represented because of financial difficulties in seeking professional help. No causal inference can be made as it is a cross-sectional study.
[47]	National Population sample, Spain	Ecological study	(2005–2010)	Inter-time Variables Pre- and post-recession period	Suicidal Behaviours National suicide rates	An 8.0 % increase was found in the suicide rate above the trend since the financial crisis (95 % CI: 1.009–1.156; *P* = 0.03). Stratified analyses suggested that the association between the crisis and suicide rates is greatest in males and those of working age.	Uses stratified analyses and adjusted for seasonal fluctuations.	Because of its ecological nature, the quality of the data is not assessable and no implications can be drawn regarding causality. The results should be interpreted with caution as other variables, independent of economic conditions, may be involved in the precipitation of suicide.
[48]	Regional population sample, Andalusia, Spain	Ecological study	24,380 (2003–2012)	Inter-time Variables Pre- and post-recession period	Suicidal Behaviours Hospital records on suicide attempts	Compared to the historical trends prior to the onset of the crisis, between 2008 and 2012 there were 4989 more suicide attempts (95 % CI: 1985–8013): 2017 (95 % CI: 87–3987) in men and 2972 (95 % CI: 1878–4075) in women. In men, an association between unemployment and suicidal behaviour was found.	First ad hoc study of the impact of the recession on suicide attempts in Spain based on hospital records in a large population sample.	Because of its ecological nature, the quality of the data is not assessable and no implications can be drawn regarding causality. The results should be interpreted with caution as other variables, independent of economic conditions, may be involved in the precipitation of suicide.
[49]	Regional population sample, Andalusia, Spain	Ecological study	1975–2012	Inter-time Variables Pre- and post-recession period	Suicidal Behaviours Regional suicide rates	Suicide rates have increased since 1975.in recent decades, an upward trend has been observed in young people (15 to 44 y), an annual percentage rate change of 1.21 (95%CI: 0.7–1.7) for men and 0.93 (95 % CI: 0.4–1.4) for women.	Regional trend analysis of the variation in suicide rates.	Because of its ecological nature, the quality of the data is not assessable and no implications can be drawn regarding causality. The results should be interpreted with caution as other variables, independent of economic conditions, may be involved in the precipitation of suicide.
[50]	National population sample, Italy	Ecological study	(1980–2010) >15 y	Inter-time Variables Pre- and post-recession period	Suicidal Behaviours National suicide rates	The suicide rate for men involved in the labour force increased by 12 % in 2010 compared with that in 2006. The suicide rate declined for women of all ages and for men younger than 25 and older than 65 years of age.	Examined trends in the total official suicide rate before and after the onset of the recession.	Because of its ecological nature, the quality of the data is not assessable and no implications can be drawn regarding causality. The results should be interpreted with caution as other variables, independent of economic conditions, may be involved in the precipitation of suicide. There were no economic variables involved in the analyses.
[51]	National population sample, England, UK	Ecological study	2008–2010	Inter-time Variables Macroeconomic indicators Pre- and post-recession period Regional unemployment rate	Suicidal Behaviours National suicide rates	During 2008 and 2010, there were 846 more (95 % CI: 818–877) suicides among men and 155 (121–189) more suicides among women than would have been expected on the basis of historical trends. The 10 % increase in men’s unemployment was significantly associated with an increase of 1.4 % (0.5–2.3 %) in suicides.	Examined trends in the total suicide rate before and after the onset of the recession and in relation to unemployment rates.	Because of its ecological nature, the quality of the data is not assessable and no implications can be drawn regarding causality. The results should be interpreted with caution as other variables, independent of economic conditions, may be involved in the precipitation of suicide.
[52]	National population sample, England and Wales, UK	Ecological study	(2001–2011) 16–64 y	Inter-time Variables Pre- and post-recession period	Suicidal Behaviours National Suicide Rates	The downward trend in the suicide rate for men stopped for men aged 16–34 years in 2006 (95 % CI Quarter 3 (Q3) 2004, Q3 2007 for 16–24-year-olds & Q1 2005, Q4 2006 for 25–34-year-olds). The suicide rate in 35–44-year-old men reversed from a downward to an upward trend in early 2010 (95 % CI Q4 2008, Q2 2011). No clear evidence of an association between trends in female suicide rates and indicators of economic recession was found.	Used age- and sex-specific trends in suicide in the years before and after the economic recession of 2008 in relation to a variety of indicators of recession effects. Excluded accidental deaths	Because of its ecological nature, the quality of the data is not assessable and no implications can be drawn regarding causality. The results should be interpreted with caution as other variables, independent of economic conditions, may be involved in the precipitation of suicide.
[53]	Cross-national population samples, EU, Canada and USA	Ecological study	2001–2011	Inter-time Variables Pre- and post-recession period	Suicidal Behaviours National suicide rates	In the EU, there was a rise in the suicide rate of 6.5 % above past trends in 2009. In Canada, suicides rose by 4.5 % between 2007 and 2009. In the USA, suicides rose by 4.8 % between 2007 and 2010.	Cross-national analysis. Examined trends in the total official suicide rate before and after the onset of the recession.	Because of its ecological nature, the quality of the data is not assessable and no implications can be drawn regarding causality. The results should be interpreted with caution as other variables, independent of economic conditions, may be involved in the precipitation of suicide. Suicide rates may vary across nations for cultural reasons.
[54]	National population sample, USA	Ecological study	1999–2010	Inter-time Variables Macroeconomic indicators Pre- and post-recession period State-level unemployment rate	Suicidal Behaviours National suicide rate	The suicide rate accelerated after the onset of the recession. There were an additional 0.51 deaths per 100,000 per year (95 % CI 0•28–0•75) in 2008–10 – an additional 1580 suicides per year (95 % CI 860–2300). A 1 % rise in unemployment is associated with a 0.99 % increase in the suicide rate (95 % CI 0 · 60–1 · 38, *p* < 0 · 0001)	Examined trends in the total suicide rate before and after the onset of the recession and in relation to unemployment rates.	Because of its ecological nature, the quality of the data is not assessable and no implications can be drawn regarding causality. The results should be interpreted with caution as other variables, independent of economic conditions, may be involved in the precipitation of suicide.

A longitudinal study from Greece showed that mental health and self-rated health were negatively affected by unemployment during the economic recession (2008–2013), especially among unemployed individuals [28]. A similar result was found in Italy, where the inequalities regarding self-reported health between workers and unemployed individuals were amplified after the onset of the recession [29].

Repeated cross-sectional studies from Greece also showed that the recession period was associated with a significant deterioration of the population’s self-reported health and increased odds of poor health when compared to control populations [30, 31]. English and Spanish repeated cross-sectional studies indicated that the prevalence of psychological distress significantly increased during the recession period, with a greater impact on men compared to women [32, 33]. However, women also reported increased mental distress during the recession, according to a repeated cross-sectional survey from Sweden [34]. In Japan, comparable surveys before and after the economic recession period showed reports of an increase in poor health across people of all socioeconomic ranks [35].

### Pre and post-economic recession changes in rates of common mental disorders

Regarding morbidity rates for common mental disorders, longitudinal data from Iceland presented aggravated stress levels among the population, though only significant for women and especially if unemployed [36].

Greek comparable data from before and after the recession exhibited a statistically significant rise in the prevalence of depression [37, 38]. In Spain, evidence displayed a risk of suffering from depression during a recession that was almost three times higher than before [39]. Similar evidence was also found in Canada and Hong Kong [40, 41]. The same Spanish study also showed an increase in the prevalence of anxiety disorders [39]. Nevertheless, no changes in the prevalence of anxiety were found in the Canadian working population sample [40].

### Pre and post-economic recession changes in substance-related disorders

A cohort study from the USA stated that the overall prevalence of any alcohol use significantly declined during the recession but, conversely, binge-drinking became more frequent [42]. Spanish repeated cross-sectional evidence shows that this recession may have triggered alcohol-related disorders, since a noteworthy rise of 4.6 % in the abuse of alcohol and dependence on it was observed [39]. Furthermore, available data from Argentina also revealed that people may tend to increase their intake of lower-quality alcohol, which is known to pose additional threats to health [43].

### Pre and post-economic recession changes in suicidal behaviours

Longitudinal evidence during the Swedish recession reported a post-recessionary increase in suicide rates among unemployed men [44] suggesting possible delayed effects of the recession on suicidal behaviours. Similarly, Greek cross-sectional data, from before and after the onset of the recession, indicated that the rate of suicidal ideation increased significantly in men [45]. Moreover, in South Korea data from comparable surveys also showed that income gradient-related suicide behaviour was found to have increased in the years after the recession period [46].

Several ecological studies from Spain have reported a substantial growth of suicidal ideation and suicide attempts [47–49]. In Italy, an ecological study showed an evident increase in suicide rates among Italian men involved in the labour force after 2007 [50]. Similar evidence comes from the UK, where time-trends analysis displayed an increase in the suicide rate, especially among working-age men [51, 52]. In an ecological analysis, Reeves et al. found that most of the European countries experienced a significant rise (6.5 %) in suicide rates after the onset of the recession in 2009 [53]. The same was found in Canada (a rise of 4.5 %) and in the USA (a rise of 4.8 %) [54].

### Macroeconomic indicators associated with mental health outcomes

Data from cohort studies focusing on unemployment rates (Table 3) have shown that high unemployment rates are linked to individuals’ worsened mental well-being and higher mental distress levels [55–57]. Similar evidence was found in a cross-sectional study from the USA [58, 59].

**Table 3:** Characteristics of studies included in review relating macroeconomic indicators and mental health outcomes, 2004–2014

Study	Setting	Study design	N Year Age	Socioeconomic determinants	Mental health outcomes	Associations/Effects	Strengths	Limitations
[55]	National population sample, USA	Cohort	26,313 18–59 y	Macroeconomic indicators State level unemployment rate	Psychological Well-being Health Related Quality of Life, Mental Health Component Summary Scale - (SF-12 Health Survey)	Increases in average state unemployment rate worsen individual’s HRQL. During hard economic times mental health decreases more than physical health	Temporal order of exposures under consideration affected all participants at the same time producing stronger causal conclusions.	The effect sizes are relatively small in magnitude. The results and recommendations should not be generalized to other cohorts.
[56]	National population sample, Britain, UK	Cohort	10,264 (1991–2008) 16–65 y	Macroeconomic indicators Local area unemployment rate	Psychological Well-being Mental health distress (GHQ-12)	Mental distress levels among unemployed people are significantly higher than among their securely employed counterparts (2.20; 95 % CI:1.98–2.42). Residence in a high-unemployment area protects against distress if unemployed.	Annual data collected over a 17 year period. Temporal order of exposures, confounders, and the outcome under consideration affected all participants at the same time producing stronger causal conclusions.	Possible bias due to selection effects threat causal inference since those with poor mental health are more likely to subsequently become unemployed.
[57]	National unemployed population sample, Sweden	Cohort	1806 (1996) 1415 (1997) 19–64 y	Macroeconomic indicators Regional unemployment and vacancy rate	Psychological Well-being Mental health distress (GHQ-12)	Significant negative effects of both unemployment rate (−0.22) and vacancy rate (−5.29) on the level of mental health among the unemployed.	Dataset surveyed in two times and shows that higher municipal vacancy rates improved mental health among the unemployed	The cross-sectional result of vacancy rates by longitudinal analysis of change gives some information on this being an effect of ecological modification and not differential health-based selection.
[59]	National population sample of working-age men, USA	Repeated cross-sectional	30,000 (1997) 35,000 (2001)	Macroeconomic indicators Local area unemployment rate	Psychological Well-being Mental health distress caseness (K6)	1 percentage point increase in the local unemployment rate leads to 3.4, 3.3, 2.5, 3.5, 3.5 and 3.8 percentage point increases in responding affirmatively to sadness, hopelessness, worthlessness, restlessness, nervousness, and feelings of effort, respectively.	Gives systematic evidence of the procyclical nature of mental health, in several clusters.	Does not take into account the lagged effect of macroeconomic conditions on mental health. Its cross-sectional design removes the possibility of causal inference.
[58]	Cross-national, 40 European and Anglo-Saxon societies	Ecological study	42,275 (2000–2004) (2005–2007)	Macroeconomic indicators Employment status, GDP, income inequality	Psychological Well-being Life satisfaction	Unemployment lowers substantially the level of life-satisfaction (−0.761 to −0.785 points lower than those employed). GDP per capita and income inequality negatively influence this association.	Large cross-national sample with attention to the macroeconomic variables of countries.	Not all contexts that affect the relationship between unemployment and life-satisfaction may be placed at the national level.
[60]	Regional population sample, Asturias, Spain	Ecological study	2000–2010	Macroeconomic indicators National unemployment rate, GDP	Psychological Well-being hospital records on incidence and prevalence of mental illness	Found a negative correlation of unemployment rate with mental health care demand. Unemployment rate was associated with a decrease in both new and prevalent mental health demand.	Regionally analysis of the association between mental health care demand and the variation of the unemployment rate and GDP.	The series studied ended in 2010, just before the intense years of the crisis. Because of its ecological nature, the quality of data is not assessable and no implications on causality can be drawn. Results should be interpreted with caution.
[61]	Cross-national samples of 30 countries EU, North American and Australia	Ecological study	1960–2012	Macroeconomic indicators National unemployment rate	Suicidal Behaviours National suicide rates	Unemployment rate increase has a detrimental impact on suicide, especially in country groups with the least developed unemployment protection (eastern and southern Europe).	Large cross-national sample covering a period of 52 years.	Because of its ecological nature, the quality of data is not assessable and no implications on causality can be drawn. Results should be interpreted with caution as other variables, independent of economic conditions, may be involved in the precipitation of suicide.
[62]	Cross-national samples, 29 EU countries	Ecological study	1999–2010	Macroeconomic indicators National unemployment rate	Suicidal Behaviours National suicide rates	A 1 % increase in unemployment rates, suicide rates increase by 0.09. Male suicides increase by 0.21 (per 100,000 male inhabitants). The relationship is positive for women but not statistically significant.	Cross-national level trends analysis covering the period of recession.	Because of its ecological nature, the quality of data is not assessable and no implications on causality can be drawn. Results should be interpreted with caution as other variables, independent of economic conditions, may be involved in the precipitation of suicide.
[63]	Cross-national samples of 26 EU countries	Ecological study	1970–2007	Macroeconomic indicators National unemployment rate	Suicidal Behaviours Substance-Disorders National suicide rates National deaths by alcohol abuse	1 % increase in unemployment increases suicide at 0.79 % in ages younger than 65 years (95 % CI 0 · 16–1 · 42; 60–550 potential excess deaths [mean 310]). A more than 3 % increase in unemployment increases suicide in 4.45 % at ages younger than 65 years (95 % CI 0 · 65–8 · 24; 250–3220 potential excess deaths [mean 1740]) and 28 % deaths from alcohol (12 · 30–43 · 70; 1550–5490 potential excess deaths [mean 3500]	Large cross-national sample covering a period of 37 years.	It is limited to 2007. Because of its ecological nature, the quality of data is not assessable and no implications on causality can be drawn. Results should be interpreted with caution as other variables, independent of economic conditions, may be involved in the precipitation of suicide.
[64]	Cross-national samples of 23 EU countries	Ecological study	2000–2010	Macroeconomic indicators National unemployment rate	Suicidal Behaviours National suicide rates	A 1 % increase in unemployment rates, suicide rates increase by 34.1 %.	Cross-national level trends analysis covering the period of recession.	Because of its ecological nature, the quality of data is not assessable and no implications on causality can be drawn. Results should be interpreted with caution as other variables, independent of economic conditions, may be involved in the precipitation of suicide. The time series is limited to a decade.
[65]	Cross-national samples of 20 EU countries	Ecological study	1981–2011	Macroeconomic indicators National unemployment rate	Suicidal Behaviours National male suicide rates	Male suicide increases significantly 0.94 % with each rise in male unemployment (95 % CI: 0.51–1.36 %)	Large cross-national sample covering a period of 30 years.	Focus only in male suicide. Because of its ecological nature, the quality of data is not assessable and no implications on causality can be drawn. Results should be interpreted with caution as other variables, independent of economic conditions, may be involved in the precipitation of suicide.
[66]	Cross-national samples of 8 EU countries	Ecological study	2000–2010	Macroeconomic indicators Unemployment rate and GDP	Suicidal Behaviours National suicide rates	Rise on unemployment rates and decline GDP incresed suicide mortality (Germany +5.3 %, Portugal +5.2 %, Czech Republic +7.6 %, Slovakia +22.7 % and Poland +19.3 %). In low social spending countries, unemployment rate has a stronger effect on suicide.	Cross-national level trends analysis covering the period of recession.	The time series is limited to a decade. The ecological design does not allow for control of potential confounders or effect modifiers. Results should be interpreted with caution as other variables, independent of economic conditions, may be involved in the precipitation of suicide.
[67]	National Population sample, USA	Ecological study	1979–2004	Macroeconomic indicators Unemployment rate and GDP	Suicidal Behaviours National suicide rates	Higher unemployment rates for prime working-age (35–64) men and women are positively correlated with their higher suicide rates	Cross-state level trends analysis	The time series is limited to 2004, higher effects are expected afterwards. The ecological design does not allow for control of potential confounders or effect modifiers. Results should be interpreted with caution as other variables, independent of economic conditions, may be involved in the precipitation of suicide.
[68]	National Population sample, USA	Ecological study	1997–2010	Macroeconomic indicators Employment Rate	Suicidal Behaviours National Suicide Rates	Strong positive association between unemployment rates and total suicide rates over time. Strong explanation among the middle-aged suicides but cannot explain temporal variation in suicide rates among the young and elderly.	Examined trends in the total suicide rate and in the rate disaggregated by sex, age group and time period and include a number of important confounding factors in a multivariate analysis.	Because of its ecological nature, the quality of data is not assessable and no implications on causality can be drawn. Results should be interpreted with caution as other variables, independent of economic conditions, may be involved in the precipitation of suicide.
[69]	National population sample, USA	Ecological study	1968–2008	Macroeconomic indicators State level unemployment rate	Suicidal Behaviours State level suicide rates	A 1-percentage-point increase in the state unemployment rate was associated with 0.16 (95 % CI: 0.08, 0.24) more suicide deaths per 100,000 population. The presence of generous state unemployment benefit programs buffer the impact of unemployment rates on suicide.	State fixed-effect analysis covering 1968–2008 on suicide rates	Because of its ecological nature, the quality of data is not assessable and no implications on causality can be drawn. Results should be interpreted with caution as other variables, independent of economic conditions, may be involved in the precipitation of suicide.
[70]	National population sample, South Korea	Ecological study	2003–2011	Macroeconomic indicators National unemployment rate	Suicidal Behaviours National suicide rates	National unemployment rate was positively and significantly associated with the unemployed and employed suicide rate.	National level trends analysis covering the period of recession.	Because of its ecological nature, the quality of data is not assessable and no implications on causality can be drawn. Results should be interpreted with caution as other variables, independent of economic conditions, may be involved in the precipitation of suicide. The time series is limited to 8 years.
[71]	National population sample, Greece	Ecological study	1968–2011	Macroeconomic indicators National unemployment rate, government expenditure	Suicidal Behaviours National suicide rates	Unemployment rates and suicide rates were highly correlated (0.45). 1 % increase in unemployment of males (25–44y), increases suicide rates in 4.5 %. Austerity measures and negative economic growth also significantly increase male suicide rates.	Evaluates specific effects of fiscal austerity, among other socio-economic variables, on suicide rates over recession period.	Because of its ecological nature, the quality of data is not assessable and no implications on causality can be drawn. Results should be interpreted with caution as other variables, independent of economic conditions, may be involved in the precipitation of suicide.
[72]	National population sample, Greece	Ecological study	1991–2011	Macroeconomic indicators Unemployment rate and GDP	Suicidal Behaviours National suicide rates	Suicide rates are positively and significantly correlated with percentage of public debt in GDP and unemployment.	National level trends analysis covering the period of recession and macroeconomic fluctuations	Because of its ecological nature, the quality of data is not assessable and no implications on causality can be drawn. Results should be interpreted with caution as other variables, independent of economic conditions, may be involved in the precipitation of suicide. The time series is limited to 10 years
[73]	National population sample, Greece	Ecological study	2000–2010	Macroeconomic indicators National unemployment rate, growth rate	Suicidal Behaviours National suicide rates	The correlations between suicidal rates and unemployment and growth rate were about zero. Found no increase in suicidality in Greece during the recession and no relationship of suicidal rates with unemployment rates or growth rate.	Evaluates specific effects of unemployment and growth rates, on suicide rates over recession period.	Because of its ecological nature, the quality of data is not assessable and no implications on causality can be drawn. Results should be interpreted with caution as other variables, independent of economic conditions, may be involved in the precipitation of suicide. The time series is limited to a decade.
[74]	National population sample, England, UK	Ecological study	1993–2010	Macroeconomic indicators National unemployment rate	Suicidal Behaviours National suicide rates	The associations between unemployment rate and suicide rates were only statistically significant associations at regional level between 2008 and 2010.	National and regional level trends analysis covering the period of recession.	Because of its ecological nature, the quality of data is not assessable and no implications on causality can be drawn. Results should be interpreted with caution as other variables, independent of economic conditions, may be involved in the precipitation of suicide.
[75]	National population sample, Hungary	Ecological study	2000–2011	Macroeconomic indicators National unemployment rate	Suicidal Behaviours National suicide rates	Unemployment rates might be associated with suicidality in the general population after 3–5 years after the onset of recession (strong positive correlation at 5 years for the general population (0.78))	National level trends analysis covering the period of recession and suggesting that there is a time lag in the increase of suicide rates.	The time series is limited to a decade Because of its ecological nature, the quality of data is not assessable and no implications on causality can be drawn. Results should be interpreted with caution as other variables, independent of economic conditions, may be involved in the precipitation of suicide.
[76]	National population sample, USA	Ecological study	2005–2010	Macroeconomic indicators State level foreclosure rate	Suicidal Behaviours State level suicide rates	The foreclosure crisis has likely contributed to increased suicides (b = 0.04; *P* < .1). the effects were strongest among the middle-aged people (46–64 years: total foreclosure rate, b = 0.21; *P* < .001)	State-level analysis covering 2005–2010 on suicide state rates.	Because of its ecological nature, the quality of data is not assessable and no implications on causality can be drawn. Results should be interpreted with caution as other variables, independent of economic conditions, may be involved in the precipitation of suicide. The time series is limited to 5 years
[77]	National population sample, Italy	Ecological study	2000–2010	Macroeconomic indicators GDP per person	Suicidal Behaviours National suicide rates	The real GDP was associated with the percentage of male completed suicides due to financial problems b = 0.16, *p* = 0.05).	National level trends analysis covering the period of recession	Only male suicides were considered. The time series is limited to a decade. The ecological design does not allow for control of potential confounders or effect modifiers. Results should be interpreted with caution as other variables, independent of economic conditions, may be involved in the precipitation of suicide.

A large cross-national ecological study has shown that rises in unemployment among the population are also associated with lower life satisfaction levels, especially among unemployed individuals [58]. Despite this evidence, however, there is a recent ecological study from Spain that suggests that rises in unemployment rates were associated with a decrease in the demand for mental healthcare [60].

Recent ecological studies provide evidence of a strong positive association between unemployment rates and suicidal behaviour. A study that covered 30 countries (European, North American, and Australia) demonstrates that increases in the unemployment rate related to the recession period have a negative impact on suicide, especially in those Eastern and Southern European countries with the least developed social protection systems [61]. Similar evidence has been found in studies focusing solely on European countries [62–66] and studies performed in the USA [67–69], as well as in South Korea [70]. In Greece two studies also found strong correlations between unemployment rates and suicide [71, 72], though there is one study reporting no correlation and no increase in suicide behaviours [73]. In England, these correlations were only statistically significant at the regional level [74], and in Hungary the correlations were only strong 3 to 5 years after the onset of the recession [75]. Using other macroeconomic indicators, Houle et al. found that the state-level foreclosure rate also correlated to suicide rates in the USA [76] and an Italian study found that the decrease in GDP per person was associated with male suicide [77].

### Individual-level indicators associated with mental health outcomes

#### Unemployment

Studies demonstrate that people who lose their job during a recession are more vulnerable to the economic recession. For instance, during the Japanese economic crisis unemployed people were twice as likely to report poor health compared to controls [35]. In Hong Kong the prevalence of a major depression episode increased among the unemployed [41]. In addition, research dealing with the European recession shows a significantly higher risk of depression and mental distress among this group of people compared to the general population [28, 29, 33, 39], although Icelandic and Swedish data showed increased stress levels only for unemployed women [34, 36]. When variations in macroeconomic indicators are considered, the unemployed were also more vulnerable to mental health problems and suicidal behaviour [56, 57, 70].

Several individual-level cohort studies (Table 4) found an association between job loss and poor mental health outcomes and low life satisfaction, and suggest that this can be both a risk factor for being unemployed and its consequence [78–86]. In addition, in cross-sectional studies unemployment has also been associated with psychosomatic symptoms and psychological distress [87–90].

**Table 4:** Characteristics of studies included in review relating unemployment status and mental health outcomes, 2004–2014

Study	Setting	Study design	N Year Age	Socioeconomic determinants	Mental health outcomes	Associations/Effects	Strengths	Limitations
[78]	National population sample, USA	Cohort	1510 (1986–2002) >25 y	Individual-level indicators Employment status Socioeconomic status	Psychological Well-being Depressive symptoms (CES-D)	Job loss is linked with follow-up depressive symptoms and, occupational prestige significantly heightened this vulnerability. Unemployment status is significantly associated with depressive symptoms (r: 0.333, S.E.: 0.108)	Temporal order of exposures, confounders, and the outcome under consideration affected all participants at the same time producing stronger causal conclusions.	Difficult to distinguish truly involuntary job losses from health-related separations. Did not account for life course effects, the role of neighbourhood, or other such effects by which inequality may shape health.
[79]	National population sample, Australia	Cohort	7176 2001 20–55 y	Individual-level indicators Employment status	Psychological Well-being Mental health distress (MHI-5)	Negative correlation (r = −0.16) between unemployment and mental health across waves. Mental health is both a consequence of and risk factor for unemployment.	Uses a continuous measure of mental health symptoms. It simultaneously investigates the bi-directional effects of unemployment and mental health.	The analyses was restricted to working age population (20 to 55 years at baseline). The results and recommendations should not be generalized to other cohorts.
[80]	National population sample, Britain, UK	Cohort	14,686 (1991–2000) ≥16 y	Individual-level indicators Employment status Financial situation	Psychological Well-being Mental health distress caseness (GHQ-12)	Job loss increased risk of distress for men (OR = 3.15; 95 % CI:2.50–3.98) and women (OR = 2.60; 95 % CI:1.97–3.43). Moving to paid work reduced risk of distress for men (OR = 0.52;95 % CI: 0.41–0.68) and for women (OR = 0.68;95 % CI: 0.69–1.40). Worse off unemployed men are more distressed (OR = 4.19; 95 % CI:3.20–5.50).	Temporal order of exposures, confounders, and the outcome under consideration affected all participants at the same time producing stronger causal conclusions.	Although, subjective financial difficulty was associated with psychological distress, whether it is causal or the consequence of negative affectivity is not clear.
[81]	National population sample, New Zealand	Cohort	15,095 (2004–2009) 15–60y	Individual-level indicators Employment Status Deprivation	Psychological Well-being Mental health distress (Kessler-10 and SF-36)	Job loss decreased MH (SF-36) in 1.34 points (95 % CI −1.85 to −0.82) and increased mental distress in 0.50 points (95 % CI 0.34 to 0.67). Deprivation was associated with a 1.47 (95 % CI −1.67 to −1.28) decline in MH and a 0.57 unit (95 % CI 0.51 to 0.63) increase in mental distress.	Large sample over 5 years. Temporal order of exposures, confounders, and the outcome under consideration affected all participants at the same time producing stronger causal conclusions.	Those with poor mental health are more likely to subsequently become unemployed or experience more deprivation, so reverse causation might be possible.
[82]	National population sample, Britain, UK	Cohort	10,300 16–64y (1991–2009)	Individual-level indicators Employment Status	Psychological Well-being Mental health distress (GHQ-12)	Moving from unemployment to employment was strongly associated with an improvement in mental health −2.1 [95 % CI −2.4 to −1.7], whereas becoming unemployed was detrimental 2.5 (95 % CI 2.2–2.7).	Annual data collected over a 19-year period. Temporal order of exposures, confounders, and the outcome under consideration affected all participants at the same time producing stronger causal conclusions.	Possible bias due to selection effects threat causal inference since those with poor mental health are more likely to subsequently become unemployed.
[83]	National population sample, Britain, UK	Cohort	10,264 16–65y (1991–2007)	Individual-level indicators Employment Status	Psychological Well-being Mental health distress (GHQ-12)	Job loss significantly predicted poorer psychological well-being in comparison to those still employed (2.21; 95 %; CI: 1.99–2.43).	Annual data collected over a 16-year period. Temporal order of exposures, confounders, and the outcome under consideration affected all participants at the same time producing stronger causal conclusions.	Possible bias due to selection effects threat causal inference since those with poor mental health are more likely to subsequently become unemployed.
[84]	National population sample, Japan	Cohort	4800 (2007–2011) 20–40y	Individual-level indicators Employment Status	Psychological Well-being Mental health distress (MHI-5)	Job loss decreases mental health by 12.0 points (MHI-5) after controlling for other variables.	Temporal order of exposures, confounders, and the outcome under consideration affected all participants at the same time producing stronger causal conclusions.	Direction of causality even after controlling for individual heterogeneity, is difficult to distinguish.
[85]	National population sample, Australia	Cohort	5846 2007 > 15 y	Individual-level indicators Employment status Unemployment duration	Psychological Well-being Mental health distress (MHI-5)	Baseline mental health status predicts overall time spent unemployed. 19.1 % of those with poor mental health experience subsequent unemployment compared with 14.6 % of those with better mental health.	Temporal order of exposures, confounders, and the outcome under consideration affected all participants at the same time producing stronger causal link.	The analysis was restricted to respondents aging 20–50 years at baseline. The results and recommendations should not be generalized to other cohorts.
[86]	National population sample, Australia	Cohort	21,280 (2001–2010) ≥16 y	Individual-level indicators Employment status Number of unemployment spells	Psychological Well-being Mental well-being (SF-36)	Compared to employed people, unemployed people show a 1.64 decrease (95 % CI −2.05 to −1.23, *p* < 0.001) in mental health, and those who had two or more spells of unemployment show a 2.56 decrease (95 % CI −3.93 to −1.19, *p* < 0.001).	Large sample. Temporal order of exposures, confounders, and the outcome under consideration affected all participants at the same time producing stronger causal conclusions.	Lack of data on voluntarily or involuntary job loss (due to illness) self-reported nature of the data on mental health.
[87]	National population, Sweden	Cross-sectional	20,538 (2008) 18–85 y	Individual-level indicators Employment status	Psychological Well-being Mental health distress (GHQ-12) psychosomatic symptoms	Unemployed people had reduced psychological well-being (OR = 2.11; 95 % CI: 1.79–2.50) and more psychosomatic symptoms (OR = 1.62; 95 % CI: 1.37–1.92) compared with individuals who were employed.	Large sample. The postal survey reduces the potential bias introduced by interviewer and respondents may answer sensitive questions more honestly.	Its cross-sectional design removes the possibility of causal inference. Postal questionnaire surveys increase non-response sample bias.
[88]	Community sample, Scania, Sweden	Cross-sectional	5180 18–64 y	Individual-level indicators Employment status Psychosocial job quality	Psychological Well-being Mental health distress (GHQ-12)	People facing job strain (OR = 3.01; 95 % CI:2.26–4.02) and unemployment (OR = 5.81; 95 % CI:4.33–7.79) have significantly higher odds ratios of psychological distress.	The postal survey reduces the potential bias introduced by interviewer and respondents may answer sensitive questions more honestly.	Its cross-sectional design removes the possibility of causal inference. Postal questionnaire surveys increase non-response sample bias.
[89]	Regional sample, North West of England, UK	Cross-sectional	15,228 (2009)	Individual-level indicators Employment status Deprivation	Psychological Well-being Life satisfaction Mental well-being	Deprivation strongly linked to low LS and MWB. 17.1 % of the most deprived tertile have low LS compared to 8.9 % in the most affluent.	It identifies the characteristics of individuals most likely to suffer from poor Well-being	Its cross-sectional design removes the possibility of causal inference.
[90]	National population sample, Brazil	Cross-sectional	5000 (2003) >18 y	Individual-level indicators Employment status	Common Mental Disorders State of animus (World Health Survey)	Among women, level of education and unemployment were associated to feelings of depression and anxiety. Among males, feelings of depression were strongly associated with unemployment.	This study provides data on the negative effects of unemployment on depression and anxiety, which are important predictors of subsequent morbidity.	Its cross-sectional design removes the possibility of causal inference. Generalizing findings may be reasonably limited to the uniqueness of the Brazilian welfare system.
[91]	Cross-national samples of older adults from 13 EU countries and USA	Cohort	15,055 (2006–2010) 50–64 y	Individual-level indicators Employment status	Common Mental Disorders Depressive symptoms (EURO-D and CESD)	Unemployment was associated with 4.78 % [95 % (CI): 0.823 to 8.74 %] increase in depressive symptoms in the USA and 3.35 % (95 % CI: 0.486 to 6.22 %) increase in Europe.	Bias due to selection and reverse causality was lessen because the study distinguished job loss due to plant closures, and used individual fixed effect models.	Used two measures for depressive symptoms Euro-D for Europe and CESD for USA. However, these were normalized. The analysis was restricted to older adults (50–64y).
[92]	Cross-national sample of primary care patients from EU and Chile	Cohort	10,059 (2003–2004) 18–75 y	Individual-level indicators Employment status	Common Mental Disorders Depression caseness Composite International Diagnostic Interview (CIDI)	Job loss between baseline and 6 months compared to those employed at both times had an adj relative risk ratio for 12-month depression of 1.58 (95 % CI:0.76, 3.27). Participants with depression at baseline and 6 months compared to neither time had an OR for 6-month unemployment of 1.58 (95 % CI:0.97, 2.58).	It examines the interrelations between unemployment and clinical depression in both directions across different countries producing stronger causal conclusions.	No available data on whether employment is full time or part-time or underemployment. If unemployed adults with depression are less likely to seek medical treatment they may be under-represented in the GP-based sampling frame.
[95]	Cross-national samples of European Countries	Cross-sectional	34,395 (2001–2009) > 18 y	Individual-level indicators Employment status Education level Income and Occupation	Common Mental Disorders Anxiety Mood disorder	Unemployed showed the highest prevalence and increased risk of 12-month mental disorders. Mood disorders and anxiety were more prevalent among those receiving a low and a low-average income Northern Ireland, Portugal and Belgium were the countries with the highest risk for mental disorders.	This study examines the associations between employment status and mental health in a European representative sample. Specifies which countries are at higher risk for mental disorders.	Since data derives from different countries during a wide time interval (2001–2009) to determine the impact of the adverse economic conditions of the past few years was not possible. Participants from different countries have been exposed to different economic scenarios and the study was unable to evaluate the impact of this. No causal inference can be made due to the cross-sectional nature of the study
[93]	National population sample, England, UK	Cross-sectional	5090 ≥16 y	Individual-level indicators Employment status	Common Mental Disorders CIS-R interview: Common Mental Disorders (CMD)	Risk of CMD was significantly greater in unemployed individuals; economically inactive; not working due to physical health reasons; unable to find a suitable job among others. Individuals unemployed for less than 1 year or more than 3 years had a higher risk of CMD.	Uses a well validated scale for detection of common mental disorders.	Its cross-sectional design removes the possibility of causal inference.
[94]	National population sample, Sweden	Cross-sectional	42,448 (2004) 18–84 y	Individual-level indicators Employment status Financial strain	Common Mental Disorders Anxiety Depression (EQ-5D)	Unemployment (OR = 2.9; 95 %; CI:2.2–4.0), economic hardship (OR = 3.1; 95 %; CI:2.4–3.9 were strongly and independently related with anxiety/depression.	Large and population-based study that uses an internationally validated scale of quality of life that measures anxiety and depression.	No causal inference can be made due to the cross-sectional nature of the study.
[96]	National population of employees of collapsed major banks, Iceland	Cross-sectional	1880 (2009) >20 y	Individual-level indicators Employment status (Downsizing)	Common Mental Disorders Depression and anxiety symptoms (AOSH)	Downsizing, salary cut, and transfer to another department is associated with increased psychological distress	Nationwide sample and the inclusion of all employees of collapsed major banks in one country highly hit by the economic recession	No causal inference can be made due to the cross-sectional design and self-reported data. This sample was drawn from the collapse of banks in Iceland, so generalizing findings to other countires may be limited
[97]	National population sample, Finland	Case–control	5859 cases 74,809 controls	Individual-level indicators Employment status Socioeconomic status	Substance-Disorders Driving under the influence of drugs (DUID)	Low education, unemployment, disability pension, being divorced and living alone were the strongest individual predictors of DUID in all substance groups.	Large sample size, based on two registers ensuring good coverage and validity, increases reliability of the study. It shows that disadvantaged social background is related to driving under the influence of drugs.	Impaired drivers were over-represented: the cases were suspected and apprehended of DUID by the police. Not all people driving under the influence are caught (fewer than 10 %). The direction of causality remains unclear.
[98]	National population sample, USA	Repeated Cross-sectional	405,000 (2002–2010) >18y	Individual-level indicators Employment status	Substance-Disorders alcohol use/abuse/dependence; illicit drug use/abuse/dependence and tobacco use	Unemployed people show higher prevalence of alcohol use, illicit drug use, tobacco use, alcohol abuse or dependence, and illicit drug abuse or dependence then employed. This was before, at the start of, and during the 2009–2010 period of high unemployment.	Nationally representative sample of US adults. Strong association between substance disorders and unemployment.	Cross-sectional data does not allow tests of causality among the reported associations. Possible bias due to validity of self-reports of sensitive behaviours
[99]	National population sample, USA	Cross-sectional	5307 (2009–2010) >18y	Individual-level indicators Employment status housing payment problems	Substance-Disorders Alcohol Dependence Negative drinking consequences	Housing payment instability was associated with experiencing more negative drinking consequences and alcohol dependence symptoms. Job loss was strongly associated with alcohol problems in univariate models, but no significant associations were observed in multivariate models.	Nationally representative sample of US adults. Strong association between alcohol drinking patterns and housing instability and unemployment.	Does not preclude the possibility of reverse causation (individuals with existing alcohol problems prior to the study)
[100]	Community sample of job-seekers, Germany	Cross-sectional	7906 (2008–09) 18–64 y	Individual-level indicators Employment status Duration of unemployment	Substance-Disorders Smoking, risky drinking, illicit drug use. Self-rated health	52.4 % of the sample (53.4 % male, 33.5 years mean age) had 3 or more health risk factors. 84.8 % of the 18–24 year old long-term unemployed men were smokers. Substance use risk factors were highest among the 18–24 year olds All health risk factors were associated with lower self-rated health.	Very high proportions of individuals with health risk behaviours were found, and associations with self-rated health were confirmed in a sample of job-seeker individuals.	No causal inference can be made due to the cross-sectional design. The validity of self-reports of sensitive behaviours, such as alcohol consumption. Since the research focused job-seekers the sample included both unemployed and employed individuals. This sample was drawn from one area in Germany so generalizing findings may be limited
[101]	National population sample, South Korea	Ecological Study	1995–2005	Individual-level indicators Employment status	Substance-Disorders Alcohol-attributable mortality	Found an incidence of 20 times higher alcohol-attributable deaths rate of unemployed compared to those of non-manual workers during recession	Brings national evidence on the inequalities in the health effects of economic changes.	Did not consider accidental deaths caused directly by alcohol (eg falls). The real magnitude of social disparity in alcohol-attributable death rates may be even greater than that estimated. Social disparity in alcohol-attributable mortality cannot be said to be a result of the crisis because this was tested.
[102]	Community sample, emergency departments in Edmonton, Canada	Case–control	507 cases 200 controls (1993–94) >16 y	Individual-level indicators Employment status	Suicidal Behaviours Parasuicide	There is an association between unemployment and parasuicide (OR = 12.0; 95 % CI:6.0–23.9)	Brings strong evidence on the influence of exposure to unemployment on parasuicidal behaviour in comparison to a control group.	There is low response rate for both cases and controls.
[103]	Cross-national samples from 21 countries worldwide	Cross-sectional	108,705 (2001–07) >18y	Individual-level indicators Employment status Educational level	Suicidal Behaviours Suicidal ideation and attempts (CIDI)	12-month prevalence of suicide ideation, plans and attempts are 2.0, 0.6 and 0.3 % respectively for developed countries and 2.1, 0.7 and 0.4 % for developing countries. Risk factors for suicidal behaviours in both developed and developing countries included being a woman, low educated, low income, and being unemployed (among others).	Large cross-national epidemiological survey database	No causal inference can be made due to the cross-sectional nature of the study. Reported suicidal ideation and suicide attempts can be susceptible to recall bias or to reluctance on the part of respondents to disclose such a sensitive information.
[104]	National population sample, Australia	Cross-sectional	4697 (2007–2009) 15–64 y	Individual-level indicators Employment status	Suicidal Behaviours Death by Suicide	During 2001–10 economically inactive/unemployed males suicide at 4.62 times (RR = 4.62; 95 % CI: 4.10, 5.19; *P* < 0.001) the rate of employed men (RR = 1.00). Economically inactive/unemployed females had a suicide RR of 8.44 compared with employed females (95 % CI 7.38, 9.67; *P* < 0.001).	Best available national data and provides information on the employment status of individual suicide cases.	Possible under-reporting of suicide data and under-report of the long-term unemployed that have given up looking for work (i.e. discouraged job seekers). Lack of available data on confounding factors.
[105]	National population sample, Spain	Cross-sectional	4583 (2001–2002) >18y	Individual-level indicators Employment status	Suicidal Behaviours Suicidal ideation and attempts (CIDI)	Being unemployed or having work disability were also associated with suicidal ideation in people aged 18–49. The prevalence of suicidal ideation and attempts found in this study is similar to the one found ten years ago, before the economic crisis	Representative sample of the national population in Spain during the economic recession. The data was collected in the same way as in the ESEMED study, making it possible to compare current figures with the prevalence found before the crisis.	No causal inference can be made due to the cross-sectional nature of the study. Reported suicidal ideation and suicide attempts can be susceptible to recall bias or to reluctance on the part of respondents to disclose such a sensitive information.

Two large cohort studies showed that unemployment was associated with depressive symptoms [91, 92]. The risk of common mental disorders such as depression and anxiety was also found to be significantly greater in unemployed individuals in several cross-sectional studies [93–96].

Furthermore, a case–control study from Finland found that being unemployed was a heavy predictor of risky behaviours such as driving under the influence of drugs [97]. Cross-sectional data from the USA and Germany also discovered that unemployment was significantly related to alcohol and drug use [98–100]. Additionally, alcohol-attributable deaths rate were determined to be higher among the unemployed population during recession, says an ecological study from South Korea [101].

Suicidal behaviours were also linked to unemployment in several studies. A Canadian case control study found that unemployed individuals have a significantly increased risk of parasuicidal behaviour compared to their matched controls [102]. Likewise, in a large cross-national study, being unemployed was discovered to be a strong risk factor for suicidal ideation and attempts [103]. An Australian study also revealed that, in times of recession, unemployed males commit suicide at 4.62 times the rate of employed men and women 8.44 times more compared with employed females [104]. Also in times of recession a Spanish study states that being unemployed was found to be associated with suicidal ideation [105].

#### Precarious and insecure work

Working conditions affect mental health (Table 5). Finnish longitudinal data pinpointed mental distress as being stronger among precarious workers with high job insecurity [106]. Nevertheless, there is a Swedish cohort study that found no significant differences in the effects of job insecurity on health between temporary and permanent workers [107]. Cross-sectional data from during the recession in Italy, determined that job stress was significantly related to workers’ mental health and fear of the crisis [108]. This was supported by British evidence of an increased risk of depression and anxiety among such employees [109, 110].

**Table 5:** Characteristics of studies included in review relating job quality and security, deprivation and socioeconomic status and mental health outcomes, 2004–2014

Study	Setting	Study design	N Year Age	Socioeconomic determinants	Mental health outcomes	Associations/Effects	Strengths	Limitations
[106]	National population sample, Finland	Cohort	3449 31 y	Individual-level indicators Psychosocial job quality and Security	Psychological Well-being Mental health distress caseness (HSCL-25) Self-reports of GP	The precarious workers have more distress symptoms in comparison with permanent workers. No differences in doctor-diagnosed/treated illnesses between precarious and permanent workers.	It measures mental health and correlates with self-reports of doctor diagnosed/treated illnesses. Temporal order of exposures and confounders affected all participants at the same time producing stronger causal conclusions.	Cannot make differential analysis of health-based selection. The results and recommendations should not be generalized to other cohorts.
[107]	Regional population sample, Northern Sweden	Cohort	1071 30–42y	Individual-level indicators Psychosocial job security	Psychological Well-being Self-rated health, sleep quality and mental health	The adverse effects of job insecurity on health are present on both permanent and temporary employees.	The study has a follow-up design. Temporal order of exposures affected all participants at the same time producing stronger causal conclusions.	The results and recommendations should not be generalized to other cohorts.
[108]	Community sample of workers from private organization, Italy	Cross-sectional	1236 (2010–2011)	Individual-level indicators Psychosocial job quality and Security	Psychological Well-being Mental health distress (GHQ12)	Job stress fully mediated the relationship between fear of the crisis and mental health of the workers.	Large sample and uses a well validated scale for detection of mental distress.	Its cross-sectional design removes the possibility of causal inference. Possible response bias since those with mental distress may perceive and rate the same work environment more stressful than those without mental distress.
[109]	National Population sample, England, UK	Cross-sectional	2603 20–55 y	Individual-level indicators Employment Status Psychosocial job quality	Common Mental Disorders CIS-R interview: Common Mental Disorders (CMD)	The prevalence of mental disorders among unemployed (33.1 %) was greater than in employed (12.9 %; OR 3.34, 95 % CI 2.06–5.42, *p* < 0.001). Results were similar for those respondents in the poorest quality jobs.	Uses a well validated scale for detection of common mental disorders.	Its cross-sectional design removes the possibility of causal inference. Possible response bias since those with mental illnesses may perceive and rate the same work environment more negatively than those without a disorder.
[110]	National working population sample, UK	Cross-sectional	3581 (2007) 16–64y	Individual-level indicators Psychosocial job security Indebtedness	Common Mental Disorders Depression	Risk of depression is greater for poor job security (OR = 1.58, 95 %; CI:1.22–2.06). Adj for age and sex, job insecurity (OR = 1.86, 95 % CI:1.47–2.35) and debt (OR = 2.17, 95 % CI:1.58–2.98) were independently associated.	Large representative sample.	Its cross-sectional design removes the possibility of causal inference: job insecurity may be more frequently reported by people rendered pessimistic by a mood disorder.
[111]	National population sample, USA	Cohort	34,653 (2001–02; 04–05) ≥20 y	Individual-level indicators Household income Socioeconomic inequalities	Common Mental Disorders Substance use disorders (AUDADIS-IV)	A decrease in household income during the 2 time points was associated with an increased risk of incident mood, anxiety, or substance use disorders (adj OR = 1.30; 99 % CI:1.06–1.60)	Nationally representative sample and strong associations. Temporal order of exposures produces stronger causal conclusions.	Unable to adjust for physical health conditions that may be potential confounders.
[112]	National population sample, New Zealand	Cohort	15,340 (2002–2004/05) >25 y	Individual-level indicators Total wealth Socioeconomic inequalities	Psychological Well-being Mental health distress (Kessler-10)	High psychological distress linked to lowest wealth quintile compared with the highest (OR 3.06, 95 % CI 2.68 to 3.50). Adj for age and sex did not alter the relationship; adj for income and area deprivation attenuated the OR to 1.73 (95 % CI 1.48 to 2.04); adj baseline health status reduced the OR to 1.45 (95 % CI 1.23 to 1.71).	Strong associations between inequalities in wealth and psychological distress. Temporal order of exposures and confounders affected all participants at the same time producing stronger causal conclusions.	The socioeconomic position at baseline was not controlled. The results and recommendations should not be generalized to other cohorts.
[113]	National population sample, Britain, UK	Cohort	8185 (1991) (2003)	Individual-level indicators Indebtedness housing payment problems	Psychological Well-being Mental health distress caseness (GHQ-12)	Housing payment problems and debts have significant detrimental effects on mental Well-being. The sizes of these effects are in addition to and larger in magnitude than those associated with financial hardship.	Temporal order of exposures, confounders, and the outcome under consideration affected all participants at the same time producing stronger causal conclusions.	Generalizing findings may be reasonably limited to the UK’s welfare system in regard to housing payment problems. The results and recommendations should not be generalized to other cohorts.
[114]	Community sample, Detroit, USA	Cohort	1547 (2008) (2010)	Individual-level indicators Home foreclosure Financial hardship	Common Mental Disorders Major depression (PHQ-9) Generalized anxiety disorder (GAD-7)	Foreclosure was associated with an increased rate of major depression [incidence density ratio (IDR) 2.4, 95 %; CI:1.6–3.6] and GAD (IDR 1.9, 95 %; CI:1.4–2.6)	Establishes longitudinal associations between home foreclosure and common mental disorders producing stronger causal conclusions.	The sample is limited to a longitudinal cohort of pre-dominantly African-American adults. Because mental health problems are common among individuals at risk of foreclosure, the observed associations may result, in part, from pre-existing psychopathology.
[115]	Community sample, Wales, UK	Cross-sectional	88,623 (2003/04–2010) 18–74 y	Individual-level indicators Area income deprivation Socioeconomic inequalities	Psychological Well-being Mental health distress (MHI-5)	High neighbourhood income inequality was associated with better mental health in low-deprivation neighbourhoods (*P* = 0.036). Income inequality at regional level was significantly associated with poorer mental health (*P* = 0.012).	Uses a continuous measure of mental health symptoms. Large sampling fraction.	No data were available on individual income. Its cross-sectional design removes the possibility of causal inference.
[116]	Community sample, France	Cohort	1103 (1991–2009) 22–35 y	Individual-level indicators Socioeconomic status Level of education	Substance-Disorders tobacco, cannabis use, other illegal drug use	Low socioeconomic status was linked with higher rates of tobacco smoking [OR = 2.11, 95 % CI 1.51–2.96], cannabis use [OR = 1.75, 95 % 1.20–2.55], problematic cannabis use [OR = 2.44, 95 % CI 1.38–4.30] and other illegal drugs [OR = 2.27, 95 % CI 1.11–4.65].	Relatively large community sample of young adults. Longitudinal measures of family and juvenile characteristics obtained independently of participants’ reports of substance-use	The research focused only young adults whose parents worked in a large national company and were part of an ongoing epidemiological study. Other variables that can act as confounders were not controlled: family and peer characteristics.
[117]	National population sample, England, UK	Cross-sectional	7461 (2007) 35–54 y	Individual-level indicators Indebtedness	Suicidal Behaviours Suicidal ideation	Those in debt were twice as likely to think about suicide after controlling for socio-demographic, economic and lifestyle factors.	Representative sample. Strong association between suicidal thoughts and being in debt.	No causal inference can be made due to the cross-sectional nature of the study.

#### Debt, deprivation, and financial hardship

Several studies found socioeconomic status and indebtedness to be related to mental health. In the USA, a cohort study, indicated increased incidence of anxiety and mood disorders, and substance use disorders were strongly associated with drops in household incomes [111]. Strong causal conclusions about this matter can also be drawn on the basis of a cohort study from New Zealand that shows a high level of association between inequalities in wealth and psychological distress, stating that people reporting low levels of wealth have three times greater distress than those reporting higher levels of wealth [112]. Longitudinal data also illustrates that housing payment problems and indebtedness have a detrimental effect on mental health [113] and on the onset of depression and anxiety [114].

Income inequality at a regional level was also significantly associated with poorer mental health in a cross-sectional study completed in a community sample from Wales, UK [115]. Additionally, low socioeconomic status was related to higher rates of tobacco smoking and the use of cannabis and other illegal drugs compared to people of higher socioeconomic status in a French community-based cohort [116].

Furthermore, a cohort and a cross-sectional study from England found that people facing debt are also at higher risk of depression [110], and are twice as likely to think about suicide [117].

The previously cited studies show that during recession Greek people with serious economic difficulties had 1.33 times higher odds of developing a major depressive episode during the recession [38], and in South Korea well-off people do better in recessions in terms of the prevalence of depression, suicidal ideation, and suicide attempts [46].

#### Families, children, and older people

The literature also stated that families and children affected by socioeconomic factors might face a decline in their mental health (Table 6). Finnish longitudinal research shows that economic stress can lead to deterioration in children’s mental health, mainly through changes in family relationships and parenting quality [118]. A large cross-national study with representative data on adolescents from 31 countries found that the countries most hit by the recession (Ireland and Portugal) faced a rise in psychological health complaints (9–17 %), and this was related to the increase in unemployment rates [119].

**Table 6:** Characteristics of studies included in review focusing children and adolescents, older adults and people with mental health problems, 2004–2014

Study	Setting	Study design	N Year Age	Socioeconomic determinants	Mental health outcomes	Associations/Effects	Strengths	Limitations
[118]	Regional sample of parents and children, Southern Finland	Cohort	1149 12 y 843 mothers 30–59y 573 fathers 28–66 y	Individual-level indicators Family perceived financial strain	Psychological Well-being Parental Mental health distress caseness (GHQ-12) Child mental health	Family economic hardship creates a risk for child mental health through economic pressures and problems in parental mental health, marital interaction, and parenting even in a welfare state.	Gives information on transgenerational effect of family economic pressure on child mental health. Child mental health was reported by both parents and children, which adds to the reliability. Temporal order of exposures, confounders, and the outcome under consideration affected all participants at the same time producing stronger causal conclusions	Reporter bias is expected since mothers and fathers reported on their own mental health and parenting. Other contexts determinants such as reductions in funding in day care and schools can act as confounders. Generalizing findings is limited to the uniqueness of the Finish welfare system with extensive governmental support to families.
[119]	Cross-national samples of adolescents, 31 countries worldwide	Repeated cross-sectional	164,623 (2005–2006) 168,284 (2009–2010) 11–15y	Inter-time Variables Macroeconomic indicators Unemployment Rates	Psychological Well-being Psychological health complaints (HBSC symptom checklist)	Ireland and Portugal were the only countries facing a rise In psychological health complaints (9–17 %) with increasing unemployment (21–148 %). Youth unemployment rates in 2010 increased the likelihood of psychological health complaints.	Uses nationally representative data on adolescents from 31 countries, surveyed over two time points, before and after recession	Data derives from 2006–2010 and the crisis started in 2008 so the long term effects of the recession could not be investigated. The sample is composed only by adolescents aging 11 to 15 years old.
[120]	National population sample of adolescent, Slovenia	Cross-sectional	1815 (2010) 15y	Individual-level indicators Family Affluence Scale, perceived material welfare, family type, occupational status of parents	Psychological Well-being Mental health (KIDSCREEN-10, SDQ), Life satisfaction Feelings of depression	The adolescents who perceived to be socioeconomically worse off had 4-times higher odds (*p* < 0.001) of a low life satisfaction, a greater chance of a low quality of life, and a higher SDQ score than those who perceived to be better off (*p* < 0.001).	Uses a national representative sample and several variables to measure socioeconomic status.	Includes only 15-year-olds who are enrolled in school and does not include dropouts, who might be among the most socioeconomically underprivileged. Its cross-sectional design removes the possibility of causal inference.
[121]	National population sample of adolescents, Portugal	Cross-sectional	4877 (2010) 10–18 y	Individual-level indicators Parental employment status	Psychological Well-being Health Related Quality of Life	Having at least one parent unemployed has a statistical significant negative impact on perceptions of adolescent health.	Gives important information about the transgenerational effect of employment status.	Its cross-sectional design removes the possibility of causal inference. The study was not designed specifically to address causal links between the variables and parental employment.
[122]	Cross-national samples of children and adolescents, Denmark and Sweden	Cross-sectional	4299 2–17 y (1996)	Individual-level indicators Parental employment status	Psychological Well-being Psychosomatic symptoms	Children in families with one or both parents without paid work had an increased prevalence of recurrent psychosomatic symptoms (OR = 1.52 to 3.20)	Gives important information about the transgenerational effect of employment status.	Underreporting bias is expected as children differ in their tendency to report symptoms to their parents. Also the parents’ reports on their children can depend on their own health. Its cross-sectional design removes the possibility of causal inference.
[123]	Regional sample of adolescents, Kosice, Slovakia	Cross-sectional	2836 14–22	Individual-level indicators Parental employment status	Psychological Well-being Self-rated health Long-term well-being Health complaints	Parental long-term unemployment (especially of fathers) is negatively associated with adolescents’ subjective health. Father’s long-term unemployment was a significant predictor of moderate self-rated health and low long-term well-being among girls and boys. Mother’s long-term unemployment was negatively associated with self-rated health of girlss and long-standing illness among boys.	Gives important information about the transgenerational effect of employment status.	Lack of specific detailed information about parental unemployment (maternity leave of mothers, retirement, or invalidity of parents were considered unemployment)
[124]	Regional sample of adolescents, emergency room, Ontario, Canada	Cohort	15,739 (2002–2011) 12–17 y	Inter-time Variables Pre and Post- recession period	Suicidal behaviours Hospital records of suicide-related behaviours	The suicide-related behaviours incidence rates decreased by 30 % in boys and girls from FYs 2002/03 to 2006/07, but stopped afterwards and subsequent admissions increased.	Large sample of adolescents and examines trends in the total suicide related behaviour during recession periods.	The hospital records do not identify suicidal intent. Data is not representative of the general population. Suicide-related behaviours are complex and other variables may act as confounders.
[125]	National population sample of adolescent, USA	Repeated cross-sectional	403,457 (1997–2009) mean age 16 y	Macroeconomic indicators State level job loss	Suicidal Behaviours Suicide ideation, attempts and plans	State level unemployment during the year preceding the survey increased girls’ probability of suicidal ideation and suicide plans, but did not affect the suicide-related behaviors of boys	Uses a national representative sample and Gives important information about economic circumstances effects on adolescents risk behaviours.	It is unable to identify the pathways through which unemployment rates affect adolescents’ suicide-related behaviors.
[126]	Regional sample of older adults, Canberra/Queanbeyan, Australia	Cohort	1973 (2005–2010) mean age 66.58 y (SD = 1.5)	Inter-time Variables Individual-level indicators Pre and Post- recession period Financial security Financial hardship	Common Mental Disorders Depression Anxiety (Goldberg Scales) Self-reported health	Economic slowdown related distress is linked to greater depression symptoms at both waves 2 (t(655) = −3.44,*p* = .001) and 3 (t(662) = −4.96, *p* < .001), and greater anxiety symptoms at both waves (wave 2 - t(655) = −3.62, *p* < .001; wave 3 - t(662) = −5.15, *p* < .001).	Temporal order of exposures, confounders, and the outcome under consideration affected all participants at the same time producing stronger causal conclusions.	The analysis was restricted to older adults at baseline. Consequently, the results and recommendations should not be applied to younger cohorts. Limited period of time, the long term effects of the recession could not be investigated.
[127]	National population sample of older adults, USA	Cohort	2261 (2005–2006) (2010–2011) >57 y	Individual-level indicators Home foreclosure	Common Mental Disorders Depressive symptoms (CES-D)	Increases in neighborhood-level foreclosure was associated with an increased rate in depression in older adults. Notices of default (OR = 1.75; 95 % CI = 1.14, 2.67) and properties returning to ownership by the bank (OR = 1.62; 95 % CI = 1.06, 2.47) were associated with depressive symptoms.	Establishes longitudinal associations between home foreclosure and depressive symptoms producing stronger causal conclusions.	The mechanisms linking increases in foreclosure to depressive symptoms are not explored. The sample is limited to a longitudinal cohort of older American adults.
[128]	Cross-national working population sample, European Union countries	Cross-sectional	20,368 (2006) 20,124 (2010) 18–64 y	Inter-time Variables Macroeconomic indicators ndividual-level indicators Pre and Post- recession period Employment Status Sate level unemployment	Psychological Well-being Mental health distress (MHI-5) Mental health disorders	Following the onset of the recession, individuals with mental health problems were more vulnerable to losing their jobs [OR = 1.12, 95 % CI: 1.03–1.34] (OR: 1.12, 95 % CI: 1.03, 1.34).	Uses nationally representative data on people with and without mental health problems from 27 countries in Europe surveyed over two time points, before and after recession.	The data was collected through brief, self-reported questionnaires. Limited period of time, the long term effects of the recession could not be investigated. Its cross-sectional design removes the possibility of causal inference.

In fact, adolescents who perceived themselves as being socioeconomically worse off have a four-times higher likelihood of rating low life satisfaction and quality of life, claims a study from Slovenia [120]. In addition, children with unemployed parents have a higher prevalence of depression, higher rates of psychosomatic symptoms, and lower perceptions of psychological well-being [121–123].

Trends in a cohort of Canadian adolescents’ total suicide-related behaviour during periods of recession illustrate that the downward trends in suicidal behaviour stopped after the onset of the recession, though no increase has been reported [124]. Moreover, in the USA, repeated cross-sectional analysis before and after the onset of the crisis revealed that state-level unemployment during the year preceding the survey increased girls’ rates of suicidal ideation and suicide plans, but did not affect the suicidal behaviour of boys [125].

Studies focusing on older adults report that those facing distress related to economic slowdown and rates of home foreclosure also had greater depression and anxiety symptoms in Australian cohort studies [126, 127].

#### People with mental health problems

A cross-sectional study comparing data from 27 EU countries before and after the crisis found that individuals with mental health problems were more vulnerable to losing their employment than those without these problems. This evidence is particularly important for people already facing mental health problems because it may indicate that during a recession discriminatory attitudes towards people with chronic mental health conditions may harden, both in the job market and in society, further increasing their suffering and isolation [128].

## Discussion

In general, evidence on the impact of economic crises and recessions on mental health is accruing, but comprehensive studies are lacking. Epidemiological data comparing changes in health status before and after a recession are consistent and report negative associations with mental health and increased mental health problems. However, to measure the extension and duration of these impacts and to isolate the exact causal factors appeared to be challenging. There is a preponderance of cross-sectional and ecological studies compared to cohort or case–control studies. This causes great limitations in terms of determining causality between the recession and mental health problems. Nevertheless, the repeated cross-sectional studies helped to better estimate the changes in the population’s outcomes before and after the recession period.

In terms of geographical allocation, most of the research is being done in Europe and North America during the period of this review (2004–2014). Some of the countries hardest hit by the economic recession (Greece, Spain, and Italy) are monitoring changes in the mental health outcomes of their populations, although they are doing so mainly by using repeated cross-sectional surveys or ecological analysis. We found no specific studies from Ireland or Portugal focusing on the effects of the recession on mental health. We strongly believe that research results from these countries could contribute to a better understanding of the consequences of the recession since its impact on mental health varies greatly, depending on how austerity measures and policy responses were implemented. Additionally, there were a very limited number of studies from low and middle-income countries despite the fact that there are strong reasons to believe that these countries are likely to be heavily affected by the recession, especially because any further reductions in these countries’ already weak health budgets (mental health services in particular) is likely to be very damaging. We argue that research from these countries finds substantial barriers to publication in widely accessible journals due to possibly material and financial constraints, problems of research design and statistics and thinkable difficulty in writing in English. Thus, we argue that this under-representation of research might result in limited conclusions.

Nonetheless, the studies included in this review confirm that recession periods are feasibly associated with the increased prevalence of psychological distress and common mental disorders, substance disorders, and ultimately suicidal behaviour. Despite being limited to the validity of self-reporting, the data on alcohol misuse behaviour indicates that any increase in its prevalence may be countercyclical and related to unemployment rates. We further add that recessions might result in an increased prevalence of smoking and illicit substance use since the literature indicated this may be a coping mechanism used to help deal with unemployment and economic distress [100]. However, the impact may vary according to the profile of substance users. Recreational users may be more susceptible to cuts in income, therefore reducing abuse, while others who are more dependent may actually adopt riskier patterns of substance misuse, such as injecting or binge drinking, in order to maximise the effects of the substances they have managed to purchase [129]. Further analysis of these fields is still required.

Although reports of growing suicidal ideation and attempts in countries in recession are limited to the complexity of the phenomenon, to the cultural background, and to the quality of the data sets and self-reports, which are susceptible to recall bias, it is consistent with the previous idea that suicide is more common in areas of high socioeconomic deprivation, social fragmentation, and unemployment [4]. Futhermore, a great proportion of the evidence from this review shows that unemployment, precarious work, debt, and financial deprivation are significantly associated with mental health problems. Determinants as such are well-known driving forces for widening health inequities, and put some groups of people at higher risk of suffering the impact of the economic recession. The influence of these factors on mental health has been widely recognised in the past [15, 130, 131]. Therefore, special attention should be given to people facing economic pressure and unemployment.

Indirect data supports the view that families and children may be disproportionally affected by recession, which is consistent with the Family Stress Model [19, 20]. Many mental disorders often start in adolescence or young adulthood. Growing up in a challenging environment can put young people in a very vulnerable position [132–134]. Up-to-date evidence shows that 27 % of young Europeans aged below 18 are at risk of poverty or social exclusion and, considering the growing number of people who are unemployed and in deprivation, these are worrying indicators [135]. Failing to protect the mental health of young people and to capitalise on their energy may indicate that we will possibly face a long-lasting loss of future adult productivity [134, 136]. Unexpectedly, there is a substantial research gap on the effects of recession on families and children. A better understanding of these effects could be gained from research focusing on how job losses and economic strain affect family members.

### Research and policy implications

Summarising the data from this review gives us a global perspective and allows some hypotheses to emerge that serve as a framework for future research on economic recessions and mental health outcomes:

- it is plausible that the actual recession increased the population’s psychological distress;
- according to the evidence reviewed, periods of recession correlate with higher prevalence of common mental disorders, substance disorders, and ultimately suicidal behaviour;
- it may be possible that in order to cope with psychosocial stress people might turn to substance misuse;
- some key factor seem to make people more vulnerable to the effects of the recession: being unemployed, having a precarious work situation, facing debts and economic strain, and having a pre-existing mental illness;
- economic recession may also have a severe and long-term impact on mental health in children and young people, especially if they face stress within the family as a result of economic hardship or parental unemployment;
- some specific differences between countries and regions were found in this review. The authors hypothesise that this may be explained by the socioeconomic response policy to recession (the presence of unemployment benefits or social programmes) which could influence changes in the mental health outcomes of the populations;
- more research is needed concerning mediating factors between the determinants of a recession and mental health outcomes;
- more research from countries badly hit by the economic recession and from low and middle income countries is needed;
- the links between recession and direct effects on health seem to be very complex, and the lagged effects have not been systematically studied because of a lack of longitudinal studies and therefore a scarcity of long data series persists.

Even though the economy can shape populations’ mental wellbeing, better mental health can in turn be a major contributor to economic growth [136]. Policies and cost-effective measures may affect the extent of the risk factors faced by populations and the occurrence of mental health disorders during and after an economic recession. The World Health Organisation [4] has argued that the mental health effects of economic crises depend on action in five key areas:

- active labour market programmes
- family support programmes
- regulation of the marketing of alcoholic beverages, restrictions on their availability, and taxation
- provision of quality and equitable access to primary care for those people at high risk of mental health problems
- debt relief programmes.

### Strengths and weaknesses of this study

A language bias might be present since the review was exclusively based on English and Portuguese language research reports. Nonetheless, the potential impact of studies published in other languages in this literature review may be minimal since most of the publications in widely accessible journals are in English.

Another limitation may be the literature search time framed to last 10 years. Although it is an usual procedure [137], it could have limited the inclusion of other important works. Also, given the heterogeneity of the metrics used by the studies we were unable to use quantitative meta-analytic methods and therefore were not capable of identifying statistical patterns.

As included studies have mainly cross-sectional or ecological design, there is a limited space for establishing causal inferences. This is especially important because this gives only evidence of the rough short-term mental health outcomes related to economic recession and specific socioeconomic indicators, but there is still a lack of evidence on the longer-term consequences, particularly if the number of long-term unemployed people continues to grow and social safety nets experience further cuts.

Moreover, despite the fact that most studies are showing negative associations between the recession and levels of mental health, there may be mixed patterns (positive and negative effects of the recession) that are dependent on countries’ policies and responses adopted to deal with the recession [14]. Thus, the generalisability of the findings is considerably limited by the uniqueness of the welfare and health systems of each country and its response measures to the economic recession itself. The only way to ascertain whether the economic recession has increased the incidence of poor mental health is to intensify the gathering of empirical evidence from long-term cohort studies [138].

Notwithstanding limitations, the literature review gives a rough approximation of the consequences of the recession, showing an increasing number of people experiencing poor mental health and reporting common mental disorders such as depression and anxiety, substance-related disorders, and suicidal behaviour, which corroborates with what was found in other reference works [1–3].

## Conclusions

Quality evidence showing that economic recessions are possibly associated with negative mental health outcomes of populations is growing. This seems especially true for psychological wellbeing, common mental disorders, substance disorders, and suicidal behaviour, despite the fact that the mediation pathways are still undisclosed. There are groups of people that may be especially vulnerable to the effects of recessions: the unemployed, those in debt or facing financial difficulties, people with pre-existing mental health problems, and families with children. It is well known that mental disorders and substance use disorders make major contributions to the global burden of disease in high-income countries and constitute important public health problems. Since economic downturns may possibly exacerbate mental ill-health and suicide risk factors, it is a collective responsibility to take action and reduce these unbearable costs as far as possible. In times of economic constraints countries may want to consider balancing appropriate resources. Structural reforms and the implementation of available cost-effective measures to achieve health and high levels of wellbeing may contribute to a more productive economy and desirable societal assets.

### Ethical approval

None sought.

## Abbreviations

MeSH: medical subject headings
GDP: gross domestic product
